# Is Camouflaging Autistic Traits Associated with Suicidal Thoughts and Behaviours? Expanding the Interpersonal Psychological Theory of Suicide in an Undergraduate Student Sample

**DOI:** 10.1007/s10803-019-04323-3

**Published:** 2019-12-09

**Authors:** S. A. Cassidy, K. Gould, E. Townsend, M. Pelton, A. E. Robertson, J. Rodgers

**Affiliations:** 1 School of Psychology, University of Nottingham, Nottingham, NG7 2RD UK; 2 Institute of Neuroscience, Newcastle University, Newcastle upon Tyne, UK; 3 Present Address: School of Psychological, Social and Behavioural Sciences, Coventry University, Coventry, UK; 4 Centre for Innovative Research Across the Lifespan, Coventry University, Coventry, UK

**Keywords:** Autistic traits, Broader autism phenotype, Camouflaging, Masking, Suicide, Suicidality, Interpersonal psychological theory of suicide, Autism spectrum conditions

## Abstract

The current study explored whether people who camouflage autistic traits are more likely to experience thwarted belongingness and suicidality, as predicted by the Interpersonal Psychological Theory of Suicide (IPTS). 160 undergraduate students (86.9% female, 18–23 years) completed a cross-sectional online survey from 8th February to 30th May 2019 including self-report measures of thwarted belongingness and perceived burdensomeness, autistic traits, depression, anxiety, camouflaging autistic traits, and lifetime suicidality. Results suggest that camouflaging autistic traits is associated with increased risk of experiencing thwarted belongingness and lifetime suicidality. It is important for suicide theories such as the IPTS to include variables relevant to the broader autism phenotype, to increase applicability of models to both autistic and non-autistic people.

## Introduction

Suicide is the leading cause of death for young people aged between 20 and 34 years in the UK (ONS 2015). Understanding and preventing suicide is highly complex and challenging, involving a combination of biological, environmental and psychological factors (Townsend 2019; O’Connor and Nock 2014; Walter and Pridmore 2012; World Health Organization 2012). There is little research into what drives the associations between autistic traits with suicidal thoughts and behaviours, to help inform new suicide prevention strategies (Cassidy and Rodgers 2017). To address this knowledge gap, research has started to explore the applicability of suicide theories developed for the general population to autistic people,^1^ and also how such theories could incorporate new psychological constructs to increase our understanding of suicide in the general population (Cassidy, in press; Pelton and Cassidy 2017). Developing suicide theory to better understand associations between autism and autistic traits with suicidal thoughts and behaviours could benefit our understanding, prediction and ultimately prevention of suicide in both autistic and non-autistic people (Cassidy, in press).

Autistic traits are preferences and behaviours characteristic of autism that are normally distributed in the general population (Baron-Cohen et al. 2001). The number of self-reported autistic traits tend to be higher in family members of autistic people, suggesting a broader autism phenotype (Constantino and Todd 2005; Piven et al. 1997). People with high autistic traits can experience similar difficulties to people diagnosed as autistic, such as in social and communication skills, adapting to change, and sensory hyper-sensitivity (Hannant et al. 2016; Robertson and Simmons 2013; Baron-Cohen et al. 2001; Piven et al. 1997). People with high autistic traits are also significantly more likely to meet criteria for a clinical diagnosis of autism (Baghdadli et al. 2017; Wigham et al. 2019; Ruzich et al. 2015). Autistic traits are frequently explored in the context of understanding increased vulnerability in autistic and non-autistic people, particularly given that many adults remain undiagnosed (Lai and Baron-Cohen 2015) or are misdiagnosed with other conditions (Au-Yeung et al. 2018). For example, autistic traits have been associated with increased vulnerability to traumatic life events (Griffiths et al. 2019), social problem-solving ability and depression (Jackson and Dritschel 2016).

Research shows that self-reported autistic traits are associated with suicidal thoughts and behaviours (Cassidy et al. 2018c; Pelton and Cassidy 2017; Paquette-Smith et al. 2014; Takara and Kondo 2014; Cassidy et al. 2014), and 40% of adults who have attempted suicide meet the cut-off for clinical concern on a validated autism screening instrument (Richards et al. 2019). Suicide theories developed for the general population stress the importance of difficulties commonly experienced by autistic people and those with high autistic traits in the formation of suicidal intent. For example, the Interpersonal Psychological Theory of Suicide (IPTS) (Van Orden et al. 2010) argues that thwarted belongingness (*I am alone*) and perceived burdensomeness (*I am a burden*) are both required in order to experience suicidal desire. In autistic adults, self-reported loneliness (an indicator of thwarted belonging) and lack of social support (an indicator of perceived burdensomeness) each predicted suicidal thoughts through depression (Hedley et al 2018; Hedley et al. 2017). Self-reported autistic traits in a non-clinical young adult sample were associated with suicidal thoughts and behaviours through thwarted belonging and perceived burdensomeness (Pelton and Cassidy 2017). This suggests that the IPTS could help explain *why* autistic people and those with high autistic traits are more likely to experience suicidal thoughts and suicidal behaviours.

However, research suggests that autistic people experience and define constructs such as quality of life (McConachie et al. 2018) or depression (Stewart et al. 2006) differently to the general population, which can affect the measurement properties of tools designed for other groups (Cassidy et al. 2018a, b; Wigham and McConachie 2014). Consistent with these concerns, the association between depression with the constructs of the IPTS was weakest in those with the highest levels of autistic traits (Pelton and Cassidy 2017). One interpretation of this result, is that perhaps the constructs of the IPTS, as defined in the general population, might not apply as strongly to those with high autistic traits. Therefore it is important to explore whether there are other autism relevant constructs which could be added to the model.

One potentially relevant factor, and previously unexplored in suicide research, is camouflaging one’s autistic traits, in order to ‘fit in’ in social situations. Social camouflaging was originally described by autistic people, who report actively attempting to mask and compensate for their autistic traits in social situations, in an attempt to fit in better with others socially (Allely 2019; Livingston et al. 2019; Cage and Troxell-Whitman 2019; Lai et al. 2017; Hull et al. 2017). Hull et al. (2019) developed the Camouflaging Autistic Traits Questionnaire (CAT-Q), to capture the extent to which both autistic and non-autistic adults engage in three aspects of social camouflaging: (1) “compensation” for autism-related difficulties in social situations, such as using scripts and copying others from carefully watching other people; (2) “masking” one’s autistic characteristics, by constantly monitoring one’s own behaviours (e.g., eye contact, facial expression, gesture) to present a non-autistic persona to others; and (3) “assimilation”, which captures behavioural strategies used to fit in better with others (e.g., forcing oneself to interact by putting on a performance and pretending). Hull et al. (2019) found that social camouflaging was significantly associated with poor mental health and well-being, consistent with a range of previous research (Cage and Troxell-Whitman 2019; Livingston et al. 2019; Allely 2019; Leedham et al. 2019; Au-Yeung et al. 2018; Camm-Crosbie et al. 2018; Cassidy et al. 2018c; Bargiela et al. 2016; Rynkiewicz et al. 2016; Rutherford et al. 2016).

Camouflaging may be particularly relevant to the IPTS, given associations with suicidality (Cassidy et al. 2018c), and feeling a lack of acceptance in society (Cage et al. 2018) which indicates thwarted belongingness. Therefore, we hypothesised that people with high levels of autistic traits will be more likely to camouflage these traits in social situations, increasing risk of feelings of thwarted belonging and thus suicidal thoughts and behaviours. We also hypothesise that camouflaging will be more strongly associated with feelings of thwarted belonging compared to perceived burdensomeness. Given that the assimilation subscale of the CAT-Q represents key aspects more relevant to thwarted belonging (i.e., not feeling that social interactions are natural or genuine), we also hypothesise that the assimilation subscale of the CAT-Q would be more strongly associated with suicidality through thwarted belonging compared to the full camouflaging scale.

## Method

### Participants

Participants were current UK undergraduate Psychology students (*n* = 160, 86.9% female, aged 18–23 years) (Table 1), recruited from the University of Nottingham, Newcastle and Coventry Psychology departments, UK, recruited between 8th February 2019 and 30th May 2019. Students received course credit in exchange for participating in the study. There were no exclusion criteria. A majority of the 227 participants who accessed the survey completed all measures with no missing data (n = 160; 70.5%).

**Table 1:** Spearman inter-correlations between all variables

Variables	AQ	CAT-Q				PHQ-9	GAD-7	PB	TB	SBQ	Age	Sex
		Total Score	Compensation	Masking	Assimilation							
AQ	–											
CAT-Q												
Total score	.566*	–										
Compensation	.428*	.826*	–									
Masking	.309*	.763*	.525*	–								
Assimilation	.589*	.784*	.446*	.399*	–							
PHQ-9	.505*	.435*	.301*	.278*	.438*	–						
GAD-7	.436*	.515*	.299*	.367*	.547*	.779*	–					
PB	.335*	.302*	.195	.193*	.315*	.626*	.573*	–				
TB	.462*	.433*	.289*	.181*	.552*	.484*	.406*	.485*	–			
SBQ	.409*	.324*	.261*	.210*	.292*	.578*	.432*	.468*	.347*	–		
Age	− .081	− .098	− .167*	.072	− .110	.085	.094	.074	− .078	.100	–	
Sex	.021	− .096	− .173*	− .180*	.118	.035	.074	− .082	− .084	− .004	− .052	–
Mean/%	8.65	90.37	29.6688	34.7313	25.9688	8.21	7.05	9.88	23.44	1.96	19.54	86.9
SD	4.4	21.49	9.54811	7.60728	9.49506	6.326	5.596	7.414	13.041	.986	1.22	–
Range	1–25	38–154	9–53	16–52	8–55	0–25	0–21	6–42	9–57	1–4	18–23	–

*N* = 160, *Sex* % female, *AQ* autism spectrum quotient short (28-item), *CAT-Q* camouflaging autistic traits questionnaire (total score, compensation subscale, masking subscale and assimilation subscale), *PHQ-9* patient health questionnaire 9-item, *GAD-7* general anxiety disorder 7-item, *PB* perceived burdensomeness, *TB* thwarted belongingness, *SBQ* suicide behaviours questionnaire—revised item 1

*Denotes *p* < .05

### Materials

#### AQ-S

The Autism Spectrum Quotient-Short (AQ-S) (Hoekstra et al. 2011) is an abridged 28-item version of the full 50-item Autism Spectrum Quotient (AQ) (Baron-Cohen et al. 2001), which measures self-reported autistic traits. The 28 items in the AQ-S were extracted from the full AQ through an item retention procedure (Hoekstra et al. 2011). Participants rate each of the 28 questions on a four-point Likert scale between “Strongly Agree” to “Strongly Disagree”. As with a majority of other studies utilising the AQ (e.g., Baron-Cohen et al. 2001; Booth et al. 2013), the AQ-S answer categories were dichotomized into “agree”/“disagree” scores. Hence, questions endorsing an autistic trait received a score of one. Scores therefore ranged from 0 to 28, with higher scores indicating higher levels of self-reported autistic traits. A systematic review showed satisfactory evidence in support of the AQ-S factor structure, internal consistency, test–retest reliability and convergent validity as rated by a validated research tool (COSMIN) (Baghdadli et al. 2017). Using the dichotomous scoring method, scores at or above a clinical cut-off of 16 have showed acceptable sensitivity and specificity in distinguishing autistic from non-autistic adults (Booth et al. 2013). α = .748.

#### CAT-Q

The Camouflaging Autistic Traits Questionnaire (CAT-Q) is a 25-item self-report questionnaire assessing the extent to which a person engages in social camouflaging behaviours (Hull et al. 2019). Hull et al. (2019) found evidence for three factors capturing different aspects of social camouflaging, which were normally distributed in autistic and general population adults: (1) “compensation” (behaviours used to compensate for autism-related difficulties in social situations) corresponding to items 1, 4, 5, 8, 11, 14, 17 20 and 23 of the questionnaire; (2) “masking” (behaviours used to hide autistic characteristics or present a non-autistic personality to others) corresponding to items 2, 6, 9, 12, 15, 18, 21 and 24 of the questionnaire; and (3) “assimilation” (behaviours used to fit in better with others and not “stand out”) corresponding to items 3, 7, 10, 13, 16 19, 22, and 25 of the questionnaire. Participants rate each of the 25 questions on a seven-point Likert scale between “Strongly Agree” to “Strongly Disagree”. As in the original study, responses were scored between 1 and 7, with higher scores for items which endorse presence of social camouflaging behaviour (Hull et al. 2019). The CAT-Q has been validated in autistic and non-autistic adults, with equivalent factor structure between groups (Hull et al. 2019). For compensation subscale α = .864. For masking subscale α = .772. For assimilation subscale α = .9. For CAT-Q full scale α = .908.

#### INQ-15

The Interpersonal Needs Questionnaire (INQ-15) is a 15-item self-report questionnaire assessing the two interpersonal constructs of the IPTS; ‘thwarted belongingness’ and ‘perceived burdensomeness’ (Van Orden et al. 2012). The INQ-15 has strong evidence for its psychometric properties, has been validated in young adults (Van Orden et al. 2012), and has been used extensively in previous research, including associations in the broader autism phenotype (Pelton and Cassidy 2017). For thwarted belongingness subscale α = .891. For Perceived burdensomeness subscale α = .914. For INQ-15 full scale α = .91.

#### PHQ-9

The Patient Health Questionnaire-9 item (PHQ-9) (Kroenke et al. 2001) is a 9-item self-report scale used to assess severity of current depressive symptoms in line with DSM-V diagnostic criteria (APA 2013). Scores range from 0 to 27 with scores at or over 10 indicating moderate, 15 moderately severe, and 20 severe depression. A recent systematic review showed that the PHQ-9 was extensively used in general population research, with strong evidence for its psychometric properties as rated by a validated research tool (COSMIN) (Cassidy et al. 2018b). α = .903.

#### GAD-7

The Generalised Anxiety Disorder-7 item (GAD-7) (Spitzer et al. 2006), is a self-report questionnaire assessing current generalised anxiety symptoms. Scores range from 0 to 21, with scores of 10 or over indicating moderate, and 15 or over severe anxiety. The GAD-7 has demonstrated good sensitivity and specificity in detecting clinical anxiety (Spitzer et al. 2006). α = .922.

#### SBQ-R

The Suicide Behaviours Questionnaire-revised (SBQ-R) is a validated 4-item self-report questionnaire that assesses lifetime suicidal behaviour, suicide ideation over the past 12 months, threat of suicide attempt, and likelihood of suicidal behaviour in the future (Osman et al. 2001). This study employed question 1 of the SBQ-R which states: “Have you ever thought about or attempted to kill yourself?” There are six possible responses from ‘never’ to ‘I have attempted to kill myself and really hoped to die’. According to their response participants are assigned to one of four categorical groups indicating no suicidal behaviour, suicide ideation, suicide plan or suicide attempt. A recent systematic review showed that the SBQ-R had been extensively used in general population research, with moderate-strong evidence in support of a range of measurement properties, rated using a validated research tool (COSMIN) (Cassidy et al. 2018c).

#### Demographic Questions

Participants indicated their age, gender, biological birth sex, and country of origin, with a majority of participants (96.3%) from the UK (Table 1).

### Ethical Approval

The current study received ethical approval from the University of Nottingham Psychology Research Ethics Committee (Ethical Approval Number: S113).

### Procedure

Participants completed an online survey using Qualtrics. Participants were fully briefed about the nature of the research, that they could skip questions and sections of the survey that made them feel uncomfortable and were provided information about relevant support services before and after taking part in the study. After providing their consent to take part, participants completed the demographics questions, AQ-S, CAT-Q, PHQ-9, GAD-7, INQ-15 and lastly item one of the SBQ-R. Subsequently participants completed a positive mood induction procedure (rating the cuteness of kittens) which has proved effective in previous research exploring similar topics (Lockwood et al. 2018; Nittono et al. 2012), and were provided a debrief including information about further support.

#### Analytic Approach

Anonymised data were exported into SPSS version 24 for analysis. Correlations explored associations between all variables. *t*-tests were used to compare specific correlation coefficients of interest. Multiple hierarchical regressions explored (after controlling for age, gender, current depression and anxiety, and autistic traits) whether camouflaging or assimilation significantly predicted thwarted belongingness and perceived burdensomeness. Serial mediation models subsequently explored whether associations between autistic traits and lifetime suicidal behaviour were mediated by camouflaging and subsequently perceived burdensomeness or thwarted belongingness, using SPSS custom dialogue box PROCESS (Field 2009; Hayes 2013), similar to the approach of previous studies (e.g., Pelton and Cassidy 2017; Cole et al. 2013).

## Results

### Descriptive Statistics

Mean AQ score in this sample was 8.65, under the clinical cut-off for possible autism diagnosis of 16, with only 6.3% of the total sample scoring at or above this cut-off. Prevalence of lifetime suicide plan was 14.4%, with suicide attempts reported in 10.6% of the current sample, similar to prevalence rates found in general and university populations (2.5–10% suicide attempts; Pelton and Cassidy 2017; Nock et al. 2008; Kessler et al. 1999; Meehan et al. 1992). Mean PHQ-9 (8.21) was below the recommended cut-off for severe depression (20), with 6.9% of the total sample scoring at or above this cut-off. Mean GAD-7 (7.07) was also below the recommended cut-off for severe anxiety (15), with 11.9% of the total sample scoring at or above this cut-off.

The INQ-15 is designed to be non-normally distributed in the general population as it measures experiences and feelings that are rare (Van Orden et al. 2012). Therefore, as in previous similar studies (Pelton and Cassidy 2017; Cole et al. 2013), all analyses were undertaken using bootstrapping procedures, as this allows for non-normal distribution in regression and mediation analyses (Field 2009; Hayes 2013). Levels of significance, direction and strength of effects for all analyses were similar regardless of bootstrapping, and therefore non-bootstrapped results are reported for ease of interpretation.

### Are Autistic Traits, Camouflaging and Lifetime Suicidality Significantly Correlated?

Data from the INQ-15 were not normally distributed, so non-parametric Spearman correlations are reported between all variables.

All variables—autistic traits, camouflaging, depression, anxiety, thwarted belongingness, and perceived burdensomeness—were significantly correlated with lifetime suicidal behaviour (Table 1). The correlations between the camouflaging questionnaire total score and subscales (compensation, masking and assimilation) with thwarted belongingness and perceived burdensomeness appeared to vary. A *t*-test comparing the correlation coefficients confirmed this: the assimilation subscale of the camouflaging questionnaire was significantly more strongly correlated with thwarted belonging (.552) than perceived burdensomeness (.315) (*t*(157) = 3.52, *p* < .01). However, overall the camouflaging questionnaire was not significantly more strongly correlated with thwarted belonging (.433) than perceived burdensomeness (.302) (*t*(157) = 1.81, *p* > .05).

### Do Autistic Traits and Camouflaging Predict Thwarted Belongingness and Perceived Burdensomeness?

Hierarchical multiple regressions were performed with thwarted belongingness and perceived burdensomeness as the outcome variables. To statistically control for these variables, age and gender were entered in the first step. The second step included depression, anxiety, and self-reported autistic traits. The last step included either the camouflaging total score, or (in a separate regression) the assimilation subscale of the camouflaging questionnaire (Tables 2, 3).

**Table 2:** Multiple hierarchical regression with (a) camouflaging and (b) assimilation predicting thwarted belongingness

	B	SE B	β	*p* value
Step 1				
Constant	43.824	16.868		
Age	− .885	.845	− .083	.297
Sex	− 3.574	3.052	− .093	.243
Step 2				
Constant	32.002	14.212		
PHQ-9	.832	.217	.404	.001
GAD-7	.013	.247	.005	.959
AQ-S	.837	.221	.282	.001
Model (a) step 3				
Constant	22.682	14.987		
CAT-Q	.096	.052	.158	.068
Model (b) step 3				
Constant	17.347	13.390		
Assimilation	.606	.114	.442	.001

R^2^ = .122 for step 1 (*p* = .31), ΔR^2^ = .351 for step 2 (*p* < .001). Model (a) ΔR^2^ = .014 for step 3 (*p* = .07); Model (b) ΔR^2^ = .099 for step 3 (*p* < .001). *N* = 160

**Table 3:** Multiple hierarchical regression with (a) camouflaging and (b) assimilation predicting perceived burdensomeness

	B	SE B	β	*p* value
Step 1				
Constant	3.787	9.637		
Age	.352	.483	.058	.467
Sex	− .907	1.744	− .041	.604
Step 2				
Constant	6.068	7.102		
PHQ-9	.767	.108	.654	.000
GAD-7	.123	.123	.093	.319
AQ-S	− .055	.110	− .033	.619
Model(a) step 3				
Constant	10.501	7.497		
CAT-Q	− .046	.026	− .132	.082
Model (b) step 3				
Constant	7.062	7.271		
Assimilation	− .041	.062	− .053	.508

R^2^ = .073 for step 1 (*p* = .66), ΔR^2^ = .505 for step 2 (*p* < .001). Model (a) ΔR^2^ = .01 for step 3 (*p* = .082); Model (b) ΔR^2^ = .001 for step 3 (*p* = .508). *N* = 160

#### Thwarted Belongingness

In step 1, the model containing age and gender did not significantly predict thwarted belongingness (F(2,157) = 1.185, *p* = .309). In step 2, depression and self-reported autistic traits (but not anxiety), accounted for significantly more of the variance (35.1%) in thwarted belongingness (F(3,154) = 28.44, *p* < .001). In step 3 of the first model, camouflaging total scores did not account for significantly more variance in thwarted belongingness (1.4%) (F(1,153) = 3.38, *p* = .068). In step 3 of the second model, the assimilation subscale of the camouflaging questionnaire accounted for significantly more variance (9.9%) in thwarted belongingness (F(1,153) = 28.18, *p* < .001) (Table 2).

#### Perceived Burdensomeness

In step 1, the model containing age and gender did not significantly predict perceived burdensomeness (F(2,157) = .417, *p* = .66). In step 2, depression (but not self-reported autistic traits or anxiety), accounted for significantly more of the variance (50.5%) in perceived burdensomeness (F(3,154) = 52.96, *p* < .001). In step 3 of the first model, camouflaging total scores did not account for significantly more variance (1%) in perceived burdensomeness (F(1,153) = 3.05, *p* = .082). In step 3 of the second model, the assimilation subscale of the camouflaging questionnaire did not account for significantly more variance (.1%) in perceived burdensomeness (F(1,153) = .44, *p* = .51) (Table 3).

### Is the Relationship Between Autistic Traits and Lifetime Suicidal Behaviour Mediated by Camouflaging, Thwarted Belonging and Perceived Burdensomeness?

Simple linear regressions showed that autistic traits (F(1,158) = 21.935, *p* < .001), camouflaging total scores (F(1,158) = 13.821, *p* < .001), the assimilation subscale of the camouflaging questionnaire (F(1,158) = 12.14, *p* < .001), thwarted belongingness (F(1,158) = 20.034, *p* < .001) and perceived burdensomeness (F(1,158) = 45.999, *p* < .001), all significantly predicted lifetime suicidality.

#### Thwarted Belongingness

**Fig. 1:**
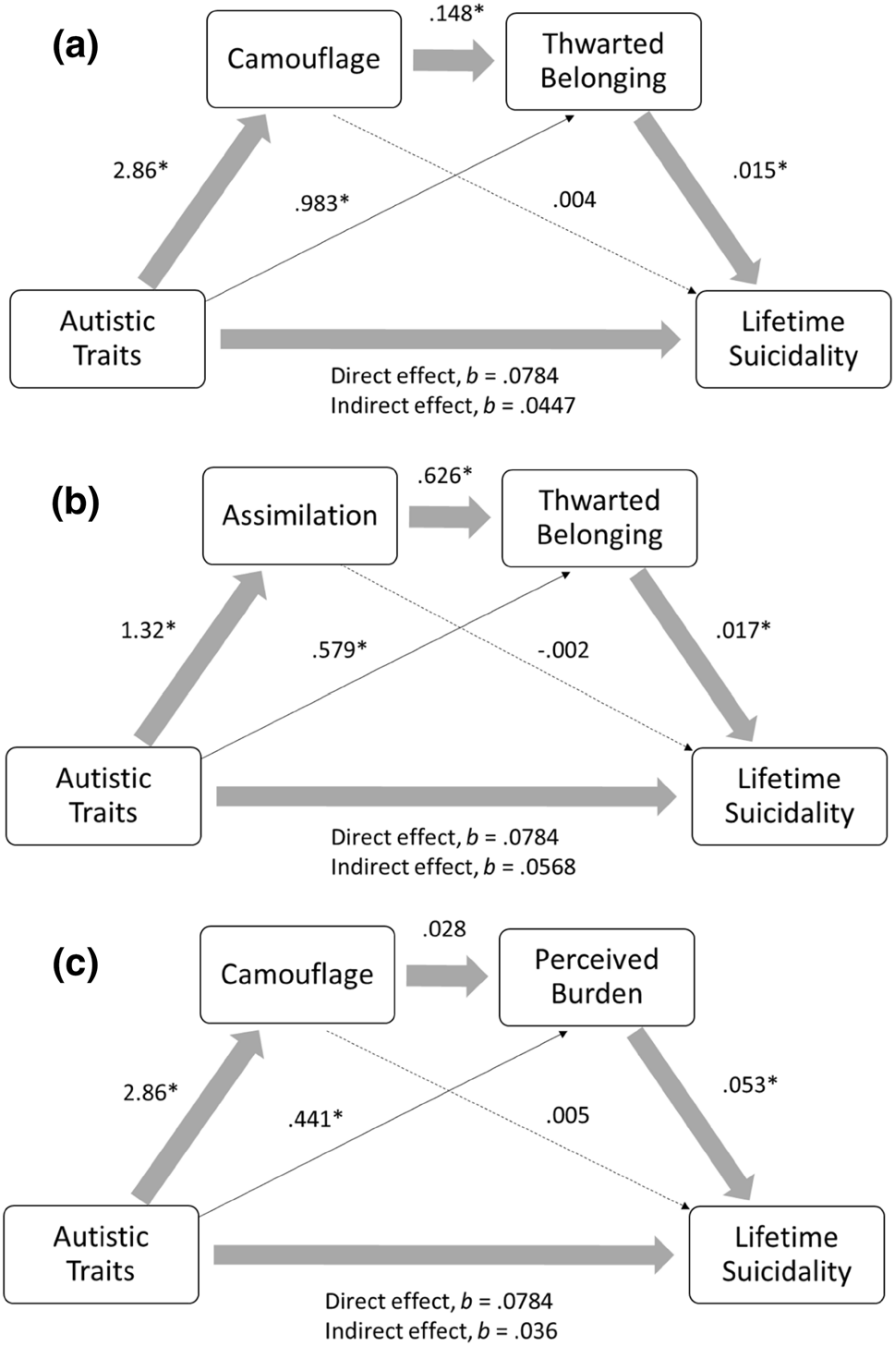
Model of the indirect effect of self-reported autistic traits on lifetime suicidal behaviour through: **a** camouflaging and thwarted belonging; **b** assimilation and thwarted belonging; and **c** camouflaging and perceived burdensomeness

There was a significant indirect effect of autistic traits on lifetime suicidal behaviour through camouflaging and thwarted belongingness (*b* = .0064 BCa CI [.001, .015]). The direct effect of autistic traits on suicidal behaviour remained significant once the mediators were added (*b* = .045, *p* = .04), indicating significant partial mediation. The path between autistic traits and suicidal behaviour was not significantly mediated by camouflaging (*b* = .012 BCa CI [− .015, .04]), but it was significantly mediated by thwarted belonging (*b* = .015 BCa CI [.001, .034]) (Fig. 1a).

There was a significant indirect effect of autistic traits on lifetime suicidal behaviour through the assimilation subscale of the camouflaging questionnaire and thwarted belongingness (*b* = .014 BCa CI [.002, .03]). The direct effect of autistic traits on suicidal behaviour remained significant once the mediators were added (*b* = .057, *p* = .008), indicating significant partial mediation. The path between autistic traits and suicidal behaviour was not significantly mediated by assimilation (*b* = − .002 BCa CI [− .033, .024]), however the path between autistic traits and lifetime suicidal behaviour was significantly mediated by thwarted belonging (*b* = .01 BCa CI [.001, .025]) (Fig. 1b).

#### Perceived Burdensomeness

The indirect effect of autistic traits on lifetime suicidal behaviour through camouflaging and perceived burdensomeness was not statistically significant (*b* = .004 BCa CI [− .011, .016]), and neither was the path between autistic traits and lifetime suicidal behaviour through camouflaging (*b* = .015 BCa CI [− .007, .041]). However, the path between autistic traits and lifetime suicidal behaviour through perceived burdensomeness was significant (*b* = .23 BCa CI [.003, .048]) (Fig. 1c).

## Discussion

The current study explored whether people with high autistic traits attempt to camouflage these traits in social situations, resulting in increased feelings of thwarted belonging, and in turn suicidal thoughts and behaviours, as predicted by the IPTS (Van Orden et al. 2010). Results from the current study support this hypothesis. The association between self-reported autistic traits with lifetime suicidal thoughts and behaviours was significantly mediated by camouflaging and thwarted belonging. Interestingly, the only non-significant path was between camouflaging and lifetime suicidal thoughts and behaviours. This suggests that camouflaging was indirectly associated with suicidal thoughts and behaviours through thwarted belonging. Camouflaging was not similarly associated with perceived burdensomeness, which significantly mediated the association between self-reported autistic traits and lifetime suicidal thoughts and behaviours.

These findings are consistent with the predictions of the IPTS (Van Orden et al. 2010) and findings from previous research. Specifically, previous studies have also shown that people with high autistic traits tend to camouflage these traits to ‘fit in’ in social situations (Hull et al. 2019), with negative consequences for mental health in autistic and non-autistic people (Hull et al. 2019), and suicidal thoughts and behaviours in autistic people (Cassidy et al. 2018c). The IPTS predicts that stable barriers to meaningful social participation lead to feelings of thwarted belonging, and subsequently suicidal intent (Van Orden et al. 2010). Previous research showed that a stable barrier to social participation (autistic traits) was significantly associated with thwarted belonging and subsequently suicidality (Pelton and Cassidy 2017). Results from the current study bring together these findings, showing that the path from autistic traits to suicidality is driven by camouflaging one’s autistic traits leading to feelings of thwarted belonging.

In line with our hypotheses, results also suggest that there is a particular component of camouflaging that is more strongly associated with thwarted belonging—assimilation. The assimilation subscale of the camouflaging scale (CAT-Q), explained significant additional variance in thwarted belonging, after controlling for age, gender, depression, anxiety and autistic traits. However, the camouflaging total score did not similarly account for significant additional variance in thwarted belonging after controlling for these factors. The association between autistic traits and lifetime suicidal thoughts and behaviours was also significantly mediated by assimilation and thwarted belonging, suggesting that those with high autistic traits, tend to try and assimilate into social situations, which results in feelings of thwarted belonging and subsequently lifetime suicidal thoughts and behaviours. The strength of the mediating effect for assimilation was larger than that for the camouflaging scale total score. The assimilation component of camouflaging therefore appears to tap into key areas which are more relevant to the construct of thwarted belonging, particularly feelings that social interactions are not natural or genuine, as you need to pretend or put on an act in social situations (Hull et al. 2019). Masking and compensation however tap into behavioural strategies and general reputation management (Hull et al. 2019), which are likely less relevant to thwarted belonging as conceptualised in the IPTS. Assimilation could therefore be considered to be a more stable barrier to achieving meaningful social connections, and thus feelings of thwarted belonging as outlined in the IPTS (Van Orden et al. 2010).

An interesting area for further study, is how specific social camouflaging is to autism, or whether this phenomenon is also present in other groups. Given that our sample did not comprise autistic people, perhaps associations between assimilation and thwarted belonging are driven by trying not to stand out from the crowd more generally, rather than camouflaging autistic traits per se. Self-reported autistic traits measured by the AQ accurately distinguish over 80% of autistic and non-autistic people (Baron-Cohen et al 2001) and predict who will later go on to obtain a diagnosis of autism (Woodbury-Smith et al 2005). However, self-reported autistic traits are also elevated in a number of other groups, such as those with psychosis (Upthegrove et al 2018) and borderline personality disorder (Dudas et al 2017). Hence, some have argued that the AQ does not specifically measure autistic traits, but more general social skills and social interest, regardless of the underlying condition (Lugnegård et al. 2015). Therefore, it is possible that camouflaging could also occur in other groups which have difficulty in assimilating into social situations, as well as those with high autistic traits or diagnosed autistic. This could be explored in future research by replicating associations between assimilation and thwarted belonging in autistic people and those from other groups (e.g., anxiety, depression, psychosis, BPD).

Results may have implications for current suicide theory, future research and clinical practice. Autism research has traditionally been plagued by the unhelpful assumption that autistic people are socially unmotivated, contrary to the testimony of autistic people (Jaswal and Acktar 2018). This has led to research exploring deficits in autistic people’s ability to accurately interpret the thoughts and feelings of non-autistic people, and clinical interventions to improve the social and communication skills of autistic people (Jaswal and Aktar 2018). However, a growing body of research is consistently showing the negative consequences of camouflaging autistic traits in social situations, demonstrating the well-intentioned but potentially damaging consequences of this traditional approach (Mitchell et al. 2019). Therefore, we must be cautious in encouraging autistic people and those with high autistic traits to modify their behaviour to fit in in social situations, and instead focus on making society more accommodating of the unique social and communication style of autistic people.

A new approach to understanding social cognition in autistic and non-autistic people is gaining empirical support. Research is showing that difficulties in social interaction between autistic and non-autistic people are two way, evidencing a ‘double empathy problem’ (Milton 2012). For example, non-autistic people have difficulty interpreting the behaviour and intentions of autistic people (Sheppard et al. 2016), which can also lead non-autistic people to rate autistic people less favourably (Alkhaldi et al. 2019). Hence, future interventions could focus on helping non-autistic people to more effectively interact with autistic people. Reducing emphasis and pressure for autistic people and those with high autistic traits to camouflage their ‘true self’ could even help prevent risk of developing mental health problems, suicidal thoughts and suicidal behaviours (Mitchell et al. 2019), and create a more useful and accurate understanding of autism that values the unique social and communication style of autistic people (Jaswal and Aktar 2018).

Our results also have implications for current suicide theory, which has not tended to include constructs relevant to autism or autistic traits, given that high rates of suicidality were not recognised in autistic people until relatively recently (Richa et al. 2014; Segers and Rawana 2014; Cassidy et al. 2014) and suicide in autism remains an under-researched area (Hedley and Uljarevic 2018; Zahid and Upthegrove 2017; Cassidy and Rodgers 2017). However, the results add to a growing body of evidence showing that autistic traits, and camouflaging autistic traits, are important risk markers for suicidal thoughts and behaviours in autistic and non-autistic people, through the constructs of the IPTS. Previous research showed that associations between depression with thwarted belonging and perceived burden were reduced at high levels of autistic traits (Pelton and Cassidy 2017), indicating that perhaps the constructs of the IPTS, as they are currently conceptualised in the general population, may not be as relevant to autism. Research is showing that constructs such as quality of life (McConachie et al. 2018) and depression (Ghaziuddin et al. 2002; Stewart et al. 2006) might be different in autistic people, with a need for new adapted measures to better capture and identify these constructs in autism (Cassidy et al. 2018b, c; Gotham et al. 2015; Wigham and McConachie 2014). Current research is therefore exploring whether the constructs of the IPTS are similarly associated with suicidal thoughts and behaviours in autistic compared non-autistic people. Another important avenue to explore will be whether thwarted belongingness and perceived burdensomeness are conceptualised the same way in autistic and non-autistic people, or whether additional autism relevant constructs (such as social camouflaging), need to be considered in relevant aspects of the model.

The current study has a number of limitations. Self-report measures of autistic traits, camouflaging, depression, anxiety, thwarted belonging and perceived burden were utilised in the current study. However, all measures have good evidence in support of their measurement properties from previous research (Hull et al. 2019; Ruzich et al. 2015; Hoekstra et al. 2011; Spitzer et al. 2006; Kroenke et al. 2001; Osman et al. 2001; Van Orden et al. 2012). Although analyses adjusted for age, sex, current depression and anxiety, additional potentially important covariates, such as socio-economic status, were not controlled for. A majority of the sample consisted of female undergraduate students. This limits the generalizability of results to the wider general population, and possibly the autistic population which has historically consisted of more males being diagnosed autistic than females (Dworzynski et al. 2012). However, autism is likely under-diagnosed in females (Dworzynski et al. 2012), in part due to camouflaging one’s autistic traits (Leedham et al. 2019; Bargiela et al. 2016; Rynkiewicz et al. 2016; Rutherford et al. 2016). Autistic females without co-occurring intellectual disability are more at risk of dying by suicide than non-autistic females (Kirby et al. 2019; Hirvikoski et al. 2016). Hence, it is important to understand why females with high autistic traits might be more likely to experience suicidal thoughts and behaviours, and the role of camouflaging in this increased risk. This study was cross-sectional, and therefore results show associations, and direction of causation cannot be confirmed.

In conclusion, results replicate and expand previous findings, showing associations between autistic traits and social camouflaging with suicidal thoughts and behaviours in autistic and non-autistic people. For the first time, results suggest that people with high autistic traits in the general population, tend to camouflage these traits in social situations, which may increase feelings of thwarted belonging and in turn risk of suicidal thoughts and behaviours. These results are consistent with the IPTS, and expand and increase the applicability of this theory to understanding and predicting suicidal thoughts and behaviours in both autistic and non-autistic people.

## Footnotes

1 We refer to “autistic people” in the current study reflecting the preferences of the autistic community (Kenny et al. 2016).
